# Fish oil-enriched nutrition combined with systemic chemotherapy for gastrointestinal cancer patients with cancer cachexia

**DOI:** 10.1038/s41598-017-05278-0

**Published:** 2017-07-06

**Authors:** Yumiko Shirai, Yoshinaga Okugawa, Asahi Hishida, Aki Ogawa, Kyoko Okamoto, Miki Shintani, Yuki Morimoto, Ryutaro Nishikawa, Takeshi Yokoe, Koji Tanaka, Hisashi Urata, Yuji Toiyama, Yasuhiro Inoue, Motoyoshi Tanaka, Yasuhiko Mohri, Ajay Goel, Masato Kusunoki, Donald C. McMillan, Chikao Miki

**Affiliations:** 1 Department of Nutrition, Iga City General Hospital, Mie, Japan; 2 Department of Surgery, Iga City General Hospital, Mie, Japan; 3 Department of Medical Oncology, Iga City General Hospital, Mie, Japan; 4 Department of Gastrointestinal and Pediatric Surgery, Division of Reparative Medicine, Institute of Life Sciences, Mie University Graduate School of Medicine, Mie, Japan; 5 Center for Gastrointestinal Research, Center for Translational Genomics and Oncology, Baylor Scott & White Research Institute and Charles A. Sammons Cancer Center, Baylor University Medical Center, Dallas, Texas USA; 6 Department of Preventive Medicine, Nagoya University Graduate School of Medicine, Nagoya, Japan; 7 Department of Nursing, Iga City General Hospital, Mie, Japan; 8 Academic Unit of Surgery, School of Medicine, University of Glasgow, Glasgow Royal Infirmary, Glasgow, UK

## Abstract

Despite recent advances in chemotherapy for gastrointestinal cancer, a crucial factor related to poor prognosis is reduced tolerance to chemotherapy induced by cancer cachexia. Fish oil (FO)-derived eicosapentaenoic acid (EPA) modulates inflammation in patients with various malignancies; however, the impact of FO-enriched nutrition as a combined modality therapy on clinical outcomes remains controversial. We systemically analysed chronological changes in biochemical and physiological status using bioelectrical impedance analysis in 128 gastrointestinal cancer patients provided with or without FO-enriched nutrition during chemotherapy. Furthermore, we evaluated the clinical significance of FO-enriched nutrition and clarified appropriate patient groups that receive prognostic benefits from FO-enriched nutrition during treatment of gastrointestinal cancer. The control group showed significant up-regulation of serum CRP) levels and no significant difference in both skeletal muscle mass and lean body mass. In contrast, the FO-enriched nutrition group showed no changes in serum CRP concentration and significantly increased skeletal muscle mass and lean body mass over time. Furthermore, high CRP levels significantly correlated with reduced tolerance to chemotherapy, and FO-enriched nutrition improved chemotherapy tolerance and prognosis, particularly in gastrointestinal cancer patients with a modified Glasgow prognostic score (mGPS) of 1 or 2. We conclude that FO-enriched nutrition may improve the prognosis of patients with cancer cachexia and systemic inflammation (i.e., those with a mGPS of 1 or 2).

## Introduction

Gastrointestinal (GI) cancer is the most common and lethal malignancy worldwide^1^. Of the top ten cancers leading to cancer-related death, five were classified as GI cancers, including oesophageal, gastric, colorectal, hepatobiliary and pancreatic cancers^2^. Furthermore, despite recent progress in surgical and medical treatments for GI cancer, these malignancies still result in 35% of all cancer deaths globally, and patients with advanced GI cancer still have a poor prognosis^2^. Treatment for GI cancer patients is critically important in the current clinical situation.

Cancer cachexia occurs in up to 80% of patients with advanced cancer^3, 4^, especially those with gastrointestinal and pancreatic malignancies, and it is a major obstacle in terms of the morbidity and mortality of patients with advanced GI cancer^5^. Cancer cachexia is defined as a “multifactorial syndrome characterized by an ongoing loss of skeletal muscle mass that cannot be fully reversed by conventional nutritional support and leads to progressive functional impairment”^3^. Accumulating evidence has suggested that systemic inflammation related to cancer is a pivotal mediator for the progression of cancer cachexia, and several pro-inflammatory cytokines released by both tumours and the host’s immune system, including interleukins (IL) 1, 2 and 6, interferon γ and TNF-α, have been implicated in the pathogenesis of cachexia in GI cancer.

Accumulating evidence has revealed a strong correlation between cancer cachexia/sarcopenia and chemotherapeutic toxicity in patients with various types of cancer^6–8^. Prado and colleagues analysed the association between the status of sarcopenia and chemotherapeutic toxicity in 55 women with advanced breast cancer resistant to anthracycline and/or taxane treatment and clarified that sarcopenia was a predictor for capecitabine toxicity in patients with metastatic breast cancer^6^. Similarly, two other studies demonstrated that decreased muscle mass was associated with an increased risk of grade 3–4 toxicity for adjuvant and palliative chemotherapy in colon cancer patients^7, 8^. Furthermore, the status of systemic inflammation could be used as a marker for predicting chemotherapeutic outcome and prognosis in patients with various types of cancers^9, 10^. Collectively, cancer cachexia, induced by the systemic inflammatory response to disease progression, increases the toxicity of chemotherapeutic treatment and exacerbates prognosis in GI cancer patients.

N-3 (omega-3) polyunsaturated fatty acids (FAs) from fish oil (FO) have plausible immune-modulating effects, partly caused by the formation of 3-series prostanoids and 5-series leukotrienes, with a low pro-inflammatory and immunosuppressive effect^11, 12^. Specifically, eicosapentaenoic acid (EPA) is a naturally occurring omega-3 fatty acid found in FO and certain marine products and has been shown to have anti-genotoxic, antioxidant, and anti-inflammatory properties through suppressing IL-6 production, down-regulating the acute phase response and reducing serum concentrations of C-reactive protein (CRP) in various types of cancer^13^. Despite evidence showing the anti-tumour and anti-cachexic activity of FO treatment in an animal model of cachexia^14^, the potential advantages of FO-enriched supplementation in nutrition parameters, quality of life, compliance with chemotherapeutic treatment, and prognosis remain controversial. These advantages have not been clarified in previous clinical trials due to several limitations, including little chronological biochemical and physiological evidence showing an alteration during nutritional intervention with FO, heterogeneous patient characteristics, little relevant nutritional support, and inadequate patient selection^15, 16^.

Previous work from our group has shown that several cytokines and serum markers reflecting the systemic inflammatory response, including IL-1b, IL-1ra, IL-6, IL-10, CRP, and albumin (Alb), are differentially expressed in serum from patients with advanced GI cancer and can be used as predictive biomarkers for postoperative nutritional status, morbidity and mortality in GI cancer patients^17–20^. To clarify the potential advantages of FO-enriched nutritional therapy in the treatment course of advanced GI cancer, we systemically investigated chronological alterations in biochemical and physiological status during chemotherapeutic treatment with or without FO-enriched nutrition in patients with advanced GI cancer. Furthermore, we analysed not only the association between FO-enriched nutritional therapy and tolerance of chemotherapeutic treatment but also the impact of FO-enriched nutrition on the long-term survival of these patients to identify appropriate subgroups eligible for FO-enriched nutrition during the treatment of advanced GI cancer.

## Results

### Serum CRP levels increased along with tumour progression in patients with GI cancer

**Figure 1:**
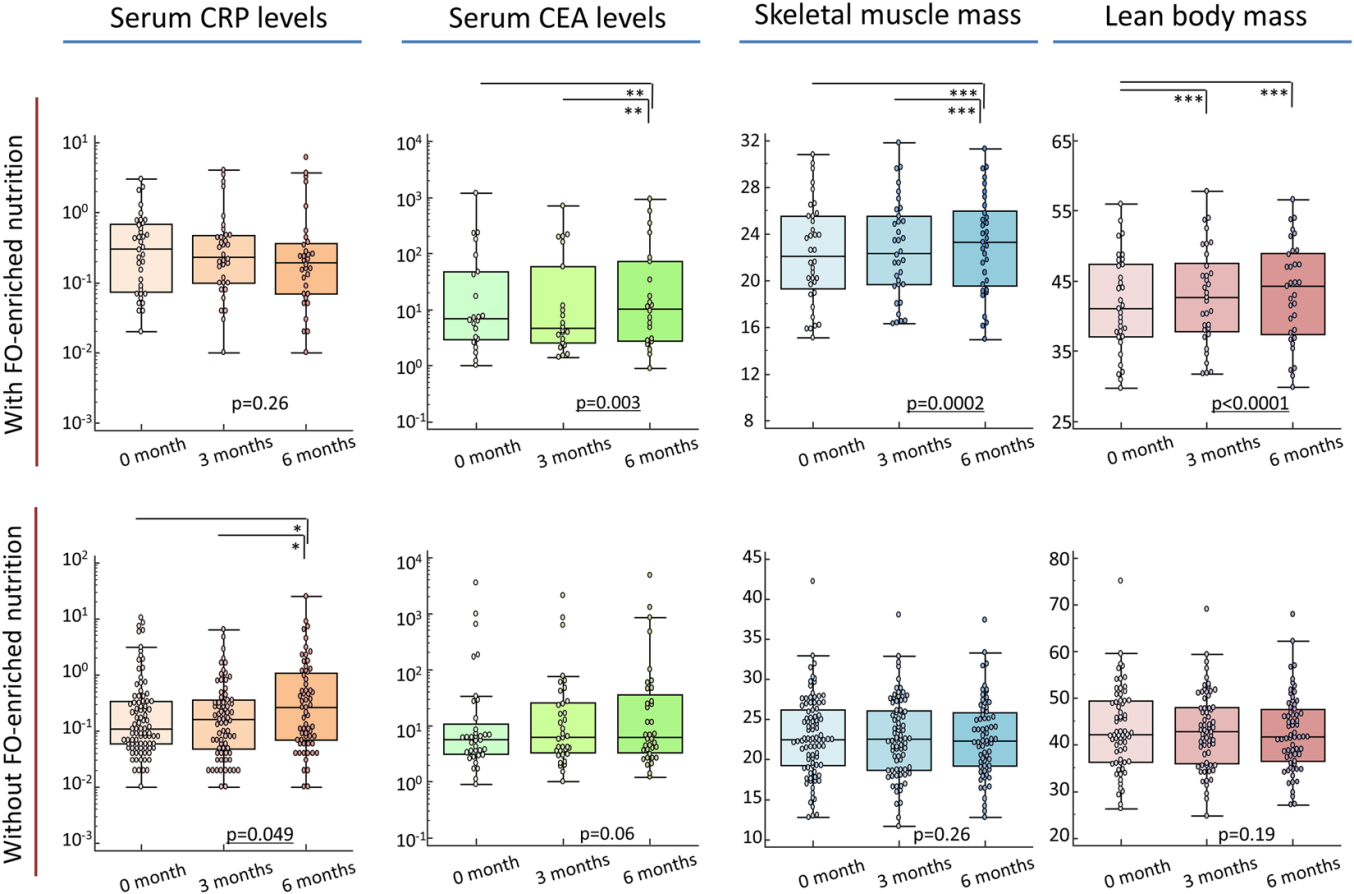
Chronological alterations in biochemical and physiological status during systemic chemotherapy in patients with GI cancer. (Upper line) Box plots are used to represent the biochemical (serum CRP and CEA levels) and physiological status (skeletal muscle mass and lean body mass) of GI patients who received FO-enriched nutrition during systemic chemotherapy. Serum CRP levels were not significantly changed in the FO-enriched nutrition group of patients with GI cancer (p = 0.26), and there was a significant improvement in the skeletal muscle mass and lean body mass of this group (p = 0.0002 and p < 0.0001, respectively). (Lower line) Box plots are used to represent the biochemical (serum CRP and CEA levels) and physiological status (skeletal muscle mass and lean body mass) of GI cancer patients not receiving FO-enriched nutrition during systemic chemotherapy. Serum CRP levels significantly increased (p = 0.049) along with serum CEA levels (p = 0.06). There were no significant differences in skeletal muscle mass or lean body mass (p = 0.26 and p = 0.19, respectively). Statistical analysis was performed using Friedman tests. All statistical tests were two-sided. *P < 0.05; **P < 0.01; ***P < 0.001.

First, we analysed the serum concentrations of CEA (carcino-embryonic antigen), carbohydrate antigen 19–9 (CA19–9) and CRP in GI cancer patients over a treatment course with or without FO-enriched nutrition therapy (Supplementary Table 1, Fig. 1). Along with tumour progression, the serum concentrations of CEA and CA19-9 significantly increased in a time-dependent manner over both treatment courses (CEA: p = 0.003, 0.06; CA19-9: p = 0.046, 0.009, Supplementary Table 1). Furthermore, serum CRP levels were positively correlated with serum CEA levels in GI cancer patients (r = 0.27, p = 0.006, Supplementary Figure 1a).

### FO-enriched nutrition inhibited increases in serum CRP levels and improved skeletal muscle mass and lean body mass in GI cancer patients

There were no significant changes in extracellular water ratio (extracellular water (ECW)/ total body water (TBW)), body fat quantity, or body fat percentage between patients treated with or without FO-enriched nutrition during the study (Supplementary Table 1). Although serum CRP levels significantly increased in a time- and disease progression-dependent manner in patients without FO-enriched nutrition (p = 0.049, Supplementary Table 1, Fig. 1), these levels remained unchanged in patients receiving FO-enriched nutrition (p = 0.26). Furthermore, in contrast to significant increases in skeletal muscle mass and lean body mass in patients with FO-enriched nutrition (p = 0.0002, p < 0.0001, respectively, Supplementary Table 1, Fig. 1), neither of these parameters showed significant changes in those not receiving FO-enriched nutrition (p = 0.26, p = 0.19, respectively).

### GI cancer patients with a modified Glasgow prognostic score of 1 or 2 showed reduced tolerance to chemotherapeutic treatment and poor prognosis

We next evaluated associations between tolerance to chemotherapy and clinicopathological factors to identify risk factors for poor tolerance to chemotherapeutic treatment. The results indicated that young age (P = 0.03) and high CRP levels (P = 0.011) correlated significantly with poor tolerance to chemotherapy (Table 1). Logistic regression analysis revealed that high CRP levels were an independent risk factor for poor tolerance to chemotherapy in this cohort (OR: 3.68, 95%CI: 1.35–10.0, p = 0.011, Table 2). Furthermore, high CRP levels were closely correlated with poor prognosis (p < 0.001, Supplementary Figure 1b). Using a Cox proportional hazards model, we demonstrated that high CRP status is an independent prognostic factor for poor overall survival (OS) in patients without FO-enriched nutrition (HR:2.88, 95%CI:1.26–6.54, p = 0.01, Supplementary Table 2). Collectively, our results indicate that the systemic inflammatory response could partly explain the poor prognosis in these patients caused by their poor tolerance to chemotherapy.

**Table 1:** Association between tolerance to chemotherapy and clinicopathological factors in GI cancer patients.

Variable		n	Chemotherapeutic treatment		*p*	
			Success (n = 79)	Failure (n = 49)		
Gender	Male	90	56	34	0.86	^*#*^
	Female	38	23	15		
Age	<71 (median)*	71	38	33	0.03*	^*#*^
	≧71	57	41	16		
Type of GI cancer	Gastrointestinal	94	57	37	0.68	^*#*^
	Hepatobiliary and pancreatic	34	22	12		
UICC stage classification	Stage II/III	44	34	10	0.01*	^*#*^
	Stage IV	84	45	39		
Serum CRP	<0.5 mg/dl**	99	67	32	0.011*	^*#*^
	≧0.5 mg/dl	29	12	17		
Serum Alb	<3.5 g/dl***	13	5	8	0.07	^*#*^
	≧3.5 g/dl	115	74	41		
FO-enriched nutrition	(+)	37	26	11	0.21	^*#*^
	(−)	91	53	38		

*The median age at treatment was 71 years in the total cohort.

^#^Chi-square test.

* ***p*** < **0.05**.

**The cut**-**off value for serum CRP level was 0.5 mg/dl.

***The cut-off value for serum Alb level was 3.5 g/dl.

**Table 2:** Multivariate analysis to identify predictors of tolerance to chemotherapeutic treatment in GI cancer patients.

Variables	Univariate			Multivariate		
	OR	95%CI	*p value*	OR	95%CI	*p value*
Gender (male)	0.93	0.43–2.03	0.86	0.87	0.37–2.04	0.74
Age (≧71 years old)^#^	0.45	0.21–0.94	0.03	0.45	0.2–1.01	0.05
Type of gastrointestinal cancer	0.81	0.56–1.17	0.26	0.69	0.45–1.05	0.09
UICC TNM stage classification	1.7	0.94–3.09	0.08	1.79	0.95–3.37	0.07
High serum CRP levels (≧0.5 mg/dl)	2.97	1.27–6.94	0.012*	3.68	1.35–10.0	0.011*
High serum Alb levels (≧3.5 g/dl)	0.35	0.11–1.13	0.08	0.38	0.1–1.51	0.17
With/without FO-enriched nutrition	0.59	0.26–1.34	0.21	0.41	0.15–1.08	0.07

^#^The median age at treatment was 71 years in this cohort. OR: Odds ratio.

* ***p*** < **0.05**.

Next, we focused on high-risk patients with elevated serum CRP levels (modified Glasgow prognostic score (mGPS) of 1 or 2) to assess whether FO-enriched nutrition could affect the tolerance to chemotherapy in these patients. Interestingly, analysis with the chi-square test revealed that tolerance to chemotherapy tended to correlate with FO-enriched nutrition in GI cancer patients with an mGPS of 1 or 2 (p = 0.05, Table 3). Based on the chronological changes in biochemical and physiological factors observed in all patients, FO-enriched nutrition may not only suppress inflammatory responses along with disease progression but also improve the nutritional status of cancer cachexia. These changes are expected to improve compliance with chemotherapeutic treatment, especially in patients with a systemic inflammatory response.

**Table 3:** Association between tolerance to chemotherapy and clinicopathological factors in patients with GI cancer and an mGPS of 1 or 2.

Variable		n	Chemotherapeutic treatment		*p*	
			Success (n = 12)	Failure (n = 17)		
Gender	Male	22	8	14	0.77	^*#*^
	Female	7	4	3		
Age	<72 (median)*	15	4	11	0.1	^*#*^
	≧72	14	8	6		
Type of GI cancer	Gastrointestinal	19	8	11	0.91	^*#*^
	Hepatobiliary and pancreatic	10	4	6		
UICC stage classification	Stage II/III	8	5	3	0.16	^*#*^
	Stage IV	21	7	14		
FO-enriched nutrition	(+)	12	8	4	0.05	^*#*^
	(−)	17	5	12		

^*^The median age at treatment was 72 years.

^#^Chi-square test.

### FO-enriched nutrition significantly improved prognosis in GI cancer patients with an mGPS of 1 or 2

**Figure 2:**
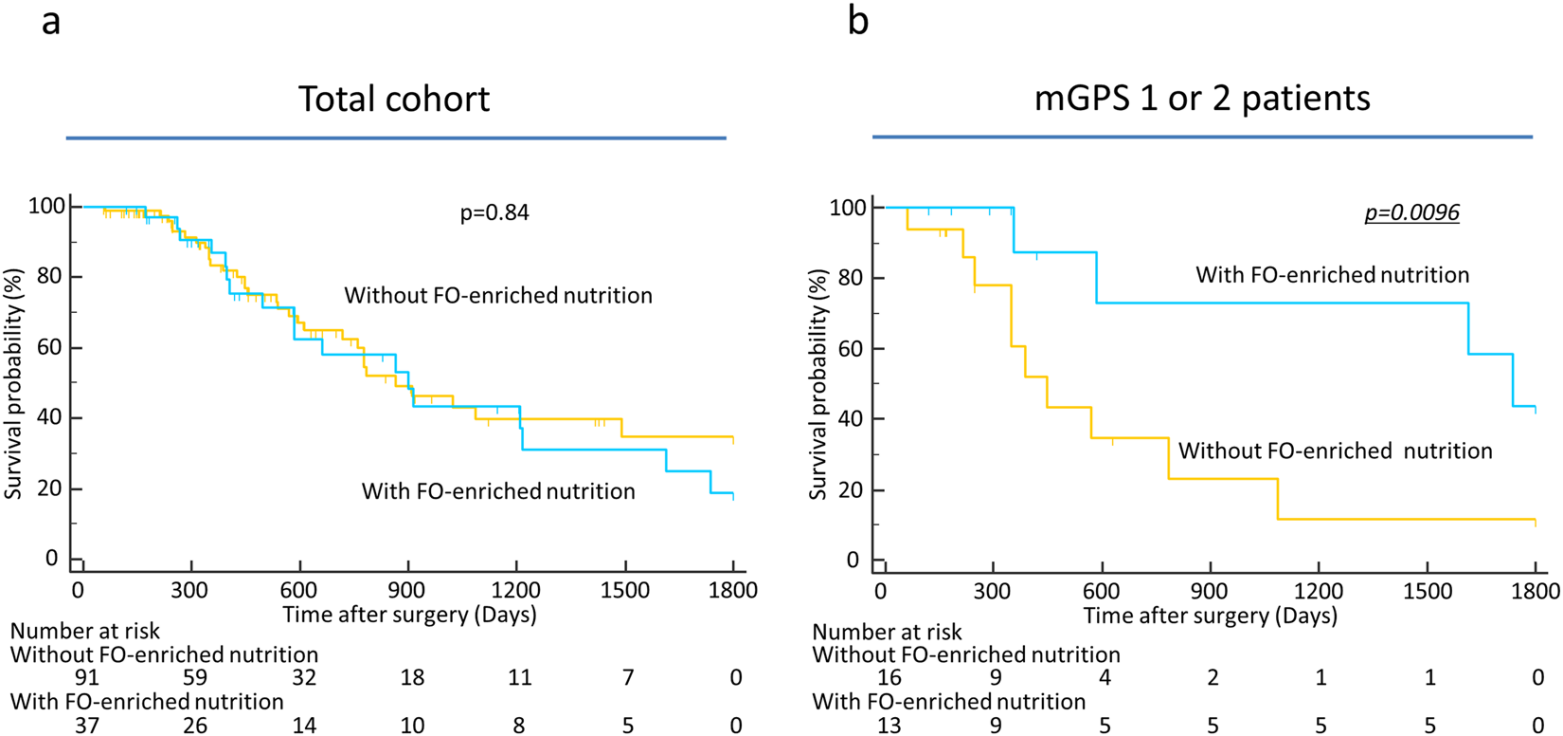
Prognostic impact of FO-enriched nutritional intervention in GI cancer patients. (**a**) Kaplan–Meier survival curves showing the OS of GI cancer patients in association with the nutritional intervention in the total cohort (n = 128). The OS rate of the GI cancer patients receiving the nutritional intervention (n = 37) did not significantly change compared with those not receiving FO-enriched nutrition (n = 91) (P = 0.84; log-rank test). (**b**) OS analysis of GI cancer patients with an mGPS of 1 or 2 based on treatment with FO-enriched nutrition. The patients with an mGPS of 1 or 2 who received FO-enriched nutrition had a better prognosis than the patients with an mGPS of 1 or 2 who did not receive FO-enriched nutrition (p = 0.0096; log-rank test). All statistical tests were two-sided.

Finally, we evaluated the prognostic impact of FO-enriched nutrition in GI cancer patients. Survival curve analysis showed no significant differences between the patient groups with or without FO-enriched nutrition among the total cohort (p = 0.84, Fig. 2a). However, the patients with an mGPS of 1 or 2 had a significantly better prognosis when they received FO-enriched nutrition (p = 0.0096, Fig. 2b). In addition, analysis using a Cox proportional hazards model demonstrated that FO-enriched nutrition is an independent predictor for improvement of prognosis in patients with an mGPS of 1 or 2 (HR:0.24, 95%CI: 0.06–0.98, p = 0.045, Table 4). Collectively, our data clearly show that FO-enriched nutrition could improve chemotherapeutic treatment compliance and prognosis in patients with an mGPS of 1 or 2.

**Table 4:** Multivariate analysis to identify predictors of overall survival in GI cancer patients with an mGPS of 1 or 2.

Variables	Univariate			Multivariate		
Gender (male)	HR	95%CI	*p value*	HR	95%CI	*p value*
	1.17	0.32–4.24	0.81	0.92	0.19–4.38	0.92
Age (≧72-year-old)^#^	0.57	0.2–1.64	0.29	0.76	0.19–2.95	0.69
Type of gastrointestinal cancer	2.02	0.91–4.49	0.08	2.39	0.86–6.62	0.09
UICC TNM stage classification	0.77	0.34–1.75	0.54	1.53	0.59–4.00	0.38
With/Without FO-enriched nutrition	0.23	0.07–0.75	0.015*	0.24	0.06–0.98	0.045*

^#^The median age at treatment was 72 years in this cohort. HR: Hazard ratio.

*p **<** ***0.05***.

## Discussion

Over the past decade, although growing evidence has supported the anti-tumour and anti-inflammatory effect of FO treatment, the clinical feasibility of FO-enriched nutrition for GI cancer patients remains controversial^15, 16^. Furthermore, the clinical feasibility of FO-enriched nutrition therapy and its impact on clinical outcomes in these patients when given during systemic chemotherapy has never been evaluated. In this study, we systematically investigated the clinical benefit of providing FO-enriched nutrition during systemic chemotherapy in a large cohort of patients with GI cancer. We made several important discoveries during this investigation. First, along with tumour recurrence and progression, the systemic inflammatory response (as reflected by serum CRP concentration) was increasingly activated during chemotherapy in patients not given FO-enriched nutrition. In contrast, the systemic inflammatory response remained stable despite disease progression in patients who received FO-enriched nutrition. Skeletal muscle mass and lean body mass remained at steady levels in patients without FO-enriched nutrition, whereas both parameters increased significantly even under systemic chemotherapy in patients who received FO-enriched nutrition. Second, we showed that patients with systemic inflammation exhibit significantly reduced tolerance to chemotherapy and that FO-enriched nutrition improved adherence to chemotherapy in GI cancer patients with an mGPS of 1 or 2. Finally, our data clearly demonstrated that FO-enriched nutrition significantly improved prognosis and was as an independent factor of improved prognosis for OS in GI cancer patients with systemic inflammation (mGPS 1 or 2).

Cachexia, which can be accompanied by skeletal muscle wasting and malnutrition, is a frequent problem in patients with malignant disease. Accumulating evidence has revealed an intimate association between cachexia and deterioration of functional status, reduced quality of life, low tolerance to chemotherapy, and poor prognosis in patients with GI cancer^21^. Furthermore, patients with advanced cancer generally develop skeletal muscle wasting, with a loss of 1.1 kg of skeletal muscle mass over the duration of chemotherapy (≥2.5 months)^22^. Based on this evidence and with recent advances in aggressive chemotherapy, oncologists are re-focusing their attention onto the management of nutritional status during systemic chemotherapy as a clinically relevant factor for supportive care. Nutritional intervention has become a widely accepted strategy for patients with cancer cachexia^23^. Furthermore, several studies have successfully demonstrated a positive effect of FO supplementation, especially with EPA, on skeletal muscle mass and lean body mass in cancer patients^24^. Fearon and colleagues conducted a double-blind, randomized, placebo-controlled study examining a large cohort of GI cancer and lung cancer patients and demonstrated a significant increase in body weight in patients receiving daily supplementation with 2 g EPA^15^. Other groups have also recently evaluated the effect of FO-derived EPA supplementation in non-small cell lung cancer (NSCLC) patients during chemotherapy^25^. In the present study, a significant reduction in whole-body skeletal muscle was observed during chemotherapy in patients without FO-enriched nutrition, whereas this chemotherapy related decline in skeletal muscle mass was prevented in patients receiving FO-enriched nutrition. Although a growing number of studies have demonstrated the potential benefits of FO and EPA supplementation for ameliorating cachexia in patients with various types of cancer, whether FO-enriched nutrition has any benefits in the clinical setting or a prognostic impact on GI cancer patients remains unclear. One key finding from our study is that FO-enriched nutrition inhibited an exaggerated systemic inflammatory response and ameliorated sarcopenia. These previous reports combined with our present data clearly suggest that FO-enriched nutrition plays a pivotal role in controlling the inflammatory response and maintaining nutritional status during chemotherapy in GI cancer patients.

Another key finding of the current study is that a high serum CRP level (mGPS 1 or 2) is an independent predictive factor for treatment interruptions or cessation of systemic chemotherapy in GI cancer patients. Moreover, FO-enriched nutrition may improve tolerance to chemotherapy and thus improve prognosis, especially in patients with systemic inflammation (mGPS 1 or 2). The clinical status of the systemic inflammatory response (based on CRP level) has been identified as an independent prognostic factor in various types of cancer, and several lines of evidence have suggested that systemic inflammation has an important role in aggravating sarcopenia. Sarcopenia is also closely correlated to poor performance status (PS)^26^, increased risk of chemotherapeutic toxicity^6^, and reduced survival in cancer patients^27^.

Interestingly, recent evidence from a randomized clinical trial comparing ruxolitinib and a placebo revealed an association between tolerance to chemotherapy and systemic inflammation status in patients with metastatic pancreatic cancer^28^. In that study, one-hundred and twenty-seven pancreatic cancer patients with distant metastasis were randomly assigned for treatment with either the Janus kinase (JAK)1/JAK2 inhibitor ruxolitinib or a placebo in conjunction with a capecitabine treatment course. Despite a non-significant difference in the prognosis of the treatment and control groups in terms of OS, benefits of ruxolitinib were seen in subgroups of patients with elevated CRP levels. Based on this evidence and the anti-inflammatory effect of FO-enriched nutrition, our findings are highly consistent with these data. Taken together, the results from the present study clearly suggest that the systemic inflammatory response might be directly involved in driving sarcopenia and poor tolerance to chemotherapy in GI cancer patients and that serum CRP levels could be used as a biomarker for selecting patients undergoing systemic chemotherapy who need FO-enriched nutrition.

Although our findings successfully demonstrated the clinical feasibility of providing an FO-enriched nutritional intervention to GI cancer patients receiving systemic chemotherapy, this study is still considered “proof of principle” and had several limitations. First, the patients receiving the intervention took FO-enriched oral nutritional supplements, whereas the patients not undergoing the intervention did not receive any additional nutritional treatment or placebo. Therefore, careful interpretation of our results is needed, and we acknowledge that the clinical feasibility and prognostic impact of FO-enriched nutrition might be affected not only by EPA nutrition but also by docosahexaenoic acid (DHA) or other nutritional components. Additionally, we performed a power calculation that estimated a minimum statistical power for our study because of our small sample size. Furthermore, we examined a single-institution, retrospective cohort. Therefore, an additional large-cohort, double-blind, multi-institutional, prospective study with multiple arms, including nutritional supplementation with EPA, nutritional supplementation with DHA, nutritional supplementation without FO, and a placebo, is needed to better clarify the clinical effect of FO and to provide supporting evidence for these novel findings. Finally, we used a bioimpedance technique to assess body composition in the current study. This method has the potential to underestimate fat mass and overestimate fat-free mass, especially in obese people and in certain ethnic groups. From this point of view, additional studies using other imaging analysis techniques to more precisely evaluate body composition may help uncover the pivotal role of FO-enriched nutrition in GI cancer patients.

In summary, our systematic and comprehensive assessment provided novel evidence for the clinical feasibility of providing FO-enriched nutrition during systemic chemotherapy for patients with GI cancer. Nutritional interventions with FO supplementation could be one component in a multimodal therapeutic approach for GI cancer, especially in patients with high serum CRP levels (mGPS 1 or 2). The present study also highlighted the need for better ways to control patient symptoms from chemotherapy to prevent discontinuation of fish oil-enriched nutrition, and thus ensure best prognosis.

## Methods

### Patients and Methods

A total of 179 patients who received therapeutic chemotherapy for advanced or recurrent GI cancer (consisting of oesophageal, gastric, colorectal, biliary and pancreatic cancers) at the Department of Medical Oncology in Iga City General Hospital (Iga, Mie, Japan) between April 2011 and April 2014 were enrolled in this retrospective cohort study. All patients had a clinical diagnosis of gastrointestinal cancer (radiological/histological/cytological confirmation), were between the ages of 18 and 80 years, and experienced a 5% or greater loss of pre-illness body weight. The patients had a life expectancy of three months or longer and a Karnofsky performance status of 70 or higher^3^. In the FO-enriched nutrition group, fifty-one patients quit the FO-enriched nutritional treatment during the study due to poor compliance and were therefore excluded. Fifty-one patients discontinued nutritional treatment, citing several reasons and circumstances related to dropping out of treatment. Discontinuation of nutritional treatment was defined when the patient stopped internal use of fish oil-enriched oral nutritional supplements for successive period sustained more than 7 days even just for once during the course of the study. The reasons for discontinuation were progressive early satiety sensation and/or dysgeusia developed as the side effects of systemic chemotherapy (58.8%), treatment-dissatisfaction (29.4%) and cost (11.8%). As a result, 128 patients were assessed during adjuvant or palliative chemotherapy (44 patients for adjuvant chemotherapy and 84 patients for palliative chemotherapy). The patients underwent measurements of body composition, including total body weight, skeletal muscle mass, body fat quantity, body fat percentage, lean body mass and extracellular/total body water, every time they visited the clinic every three months. The criterion for tolerance of systemic chemotherapy was the completion of cancer treatment as planned (defined as completing the initially planned chemotherapy course without later modifications or early discontinuation), as previously described^29^. The Tumour Node Metastasis (TNM) staging system from the American Joint Committee on Cancer was used for pathological tumour staging of gastrointestinal cancer^30^. During each hospital visit every three months, all patients underwent a chest X-ray and abdominal computed tomography. Patients who were treated with radiotherapy or chemotherapy before surgery were excluded from the study. The diagnosis of gastrointestinal cancer was confirmed for all enrolled patients based on clinicopathological findings. Patients were randomly assigned to a treatment course with or without FO-enriched nutrition by the healthcare staff. Patients in the FO-enriched nutrition group received one or two packs of FO-enriched oral nutritional supplements per day according to their tolerance for FO-enriched nutrition (Prosure®, Abbott Nutrition, Tokyo, Japan) and took the product for six months. Each pack contained 16 g of protein, 1.1 g of EPA, 0.5 g of DHA, and a total of 355 kcal. In contrast, patients who did not receive FO-enriched nutrition were not given any additional nutritional treatment or placebo. Patient characteristics are summarized in Table 5. All methods in this study were performed in accordance with the relevant guidelines and regulations. Written informed consent was obtained from all patients, and the study was approved by the Institutional Review Board of Iga City General Hospital.

**Table 5:** Patient characteristics and comparison between clinicopathological factors and treatment course in patients with GI cancer.

	With FO-enriched nutrition (n = 37)	Without FO-enriched nutrition (n = 91)	*p value*
Gender			
Male (n = 90)	26	64	0.99^#^
Female (n = 38)	11	27	
Age (mean ± SD)	72.3 ± 8.4	68.9 ± 10.3	0.08*
Type of GI cancer			
Gastrointestinal	25	69	0.34^#^
Hepatobiliary and pancreatic	12	22	
UICC Stage classification			
II/III	12	32	0.77^#^
IV	25	59	

*Student’s t-test, ^#^Chi-square test.

### Measurements

Routine laboratory measurements, including serum CRP and albumin, were carried out on the same day. No patients showed clinical evidence of infection at any measurement point. Serum CEA and CA19-9 were also routinely measured at the same time using an enzyme immunoassay on a Tosoh AIA 21 (Diamond Diagnostics, Holliston, MA, USA). Analysis of body composition, including total body weight, height, body mass index (BMI), lean body mass, fat mass, skeletal muscle mass, body fat quantity, body fat percentage, and extracellular/total body water, was conducted using bioelectrical impedance (InBody®, Cerritos, CA, USA) every three months.

The mGPS was determined as previously described^31, 32^. Briefly, patients with elevated CRP (>0.5 mg/dl) were given a score of 1 or 2 depending on the absence or presence of hypoalbuminemia (<3.5 g/dl). Patients with a normal CRP and any albumin level were given a score of 0.

### Statistical methods

Statistical analysis was performed using Medcalc version 16.4 (Broekstraat 52, 9030; Mariakerke, Belgium). The results are expressed as the mean ± standard deviation (SD). Differences between groups were estimated using the chi-square test, Student’s t-test, and the Friedman test when appropriate. F-tests were conducted to assess the equality of variance for comparable groups. For time-to-event analyses, survival estimates were calculated using Kaplan-Meier analysis, and groups were compared with the log-rank test. OS was measured from the date the patient underwent surgery to the date of death resulting from any cause or the last known follow-up for patients who were still alive. Cox’s proportional hazards model was used to estimate hazard ratios (HRs) for death. Assumption of proportionality was confirmed for the Cox proportional hazards analyses by generating Kaplan-Meier survival curves (e.g., groups with vs. without FO-enriched nutrition) and by ensuring that the two curves did not intersect with one another. Multivariate logistic regression models were used to predict factors influencing tolerance to systemic chemotherapy. Forced-entry regression was used to include these variables in all multivariable equations in order to analyse whether each of the predictors affected the outcome after adjusting for known confounders. Tests for an interaction were performed using the likelihood ratio test of cross-product terms. All P values were 2-sided, and those less than 0.05 were considered statistically significant.

### Sample size justification

With regard to the sample size used for the present study to assess the performance of prognostic markers for survival, power calculations were performed based on a detection difference of 0.05 between treatment and non-treatment groups. Based on the patient number and distribution in this study (n = 37 for the treatment group and n = 91 for the control group), we estimated a 99.9% and 100% power to substantiate a HR of 3 and 4, respectively, for predicting prognostic outcomes at a significance level of 0.05. Furthermore, focusing only on the patients with high CRP levels (n = 13 for the treatment group and n = 16 for the control group), enough estimated power (75.5% and 86.2%) to substantiate HRs of 3 and 4 could be achieved for predicting prognostic outcomes.

### Data availability

The datasets analysed during the current study are available from the corresponding author upon reasonable request.

## Electronic supplementary material

Supplementary figure, figure legend and table
