# Supplementary material for: Fish oil-enriched nutrition combined with systemic chemotherapy for gastrointestinal cancer patients with cancer cachexia
Source: Sci Rep. 2017 Jul 6;7:4826. doi: 10.1038/s41598-017-05278-0 (PMC5500468; doi:10.1038/s41598-017-05278-0)
Supplement: Supplementary file 1 — Supplementary figure, figure legend and table [file 41598_2017_5278_MOESM1_ESM.doc]

**Fish oil-enriched nutrition combined with systemic chemotherapy**

**for gastrointestinal cancer patients with cancer cachexia**

**Short title: Feasibility of fish oil-enriched nutrition in GI cancer**

Yumiko Shirai^1#^, Yoshinaga Okugawa^2,3,4,5#*^, Asahi Hishida^6^, Aki Ogawa^7^, Kyoko Okamoto^7^, Miki Shintani^1^, Yuki Morimoto^2^, Ryutaro Nishikawa^2^, Takeshi Yokoe^2^, Koji Tanaka^2^, Hisashi Urata^2^, Yuji Toiyama^4^, Yasuhiro Inoue^4^, Motoyoshi Tanaka^3^, Yasuhiko Mohri^4^, Ajay Goel^5^, Masato Kusunoki^4^, Donald C. McMillan^8^, and Chikao Miki^2*^

^1^ Department of Nutrition, Iga City General Hospital, Mie, Japan

^2^ Department of Surgery, Iga City General Hospital, Mie, Japan

^3^ Department of Medical Oncology, Iga City General Hospital, Mie, Japan

^4^ Department of Gastrointestinal and Pediatric Surgery, Division of Reparative Medicine, Institute of Life Sciences, Mie University Graduate School of Medicine, Japan

^5^ Center for Gastrointestinal Research; Center for Translational Genomics and Oncology, Baylor Scott & White Research Institute and Charles A. Sammons Cancer Center, Baylor University Medical Center, Dallas, Texas, USA

^6^ Department of Preventive Medicine, Nagoya University Graduate School of Medicine, Nagoya, Japan

^7^ Department of Nursing, Iga City General Hospital, Mie, Japan

^8^ Academic Unit of Surgery, School of Medicine, University of Glasgow, Glasgow Royal Infirmary, Glasgow, UK

**Supplementary files**

**Supplementary Figure Legend**

**Supplementary Figure 1**: **(a) Significant correlation between pretreatment serum CEA levels and CRP levels in this cohort.** Pre-treatment serum CRP levels showed a significantly positive correlation with serum CEA levels in GI cancer patients (r=0.27, p=0.006). **(b) Kaplan-Meier survival analysis of GI cancer patients without FO-enriched nutrition based on pretreatment serum CRP status.** High CRP levels in pretreatment serum were significantly correlated with poor prognosis in GI cancer patients not receiving FO-enriched nutrition (p<0.001; log-rank test).


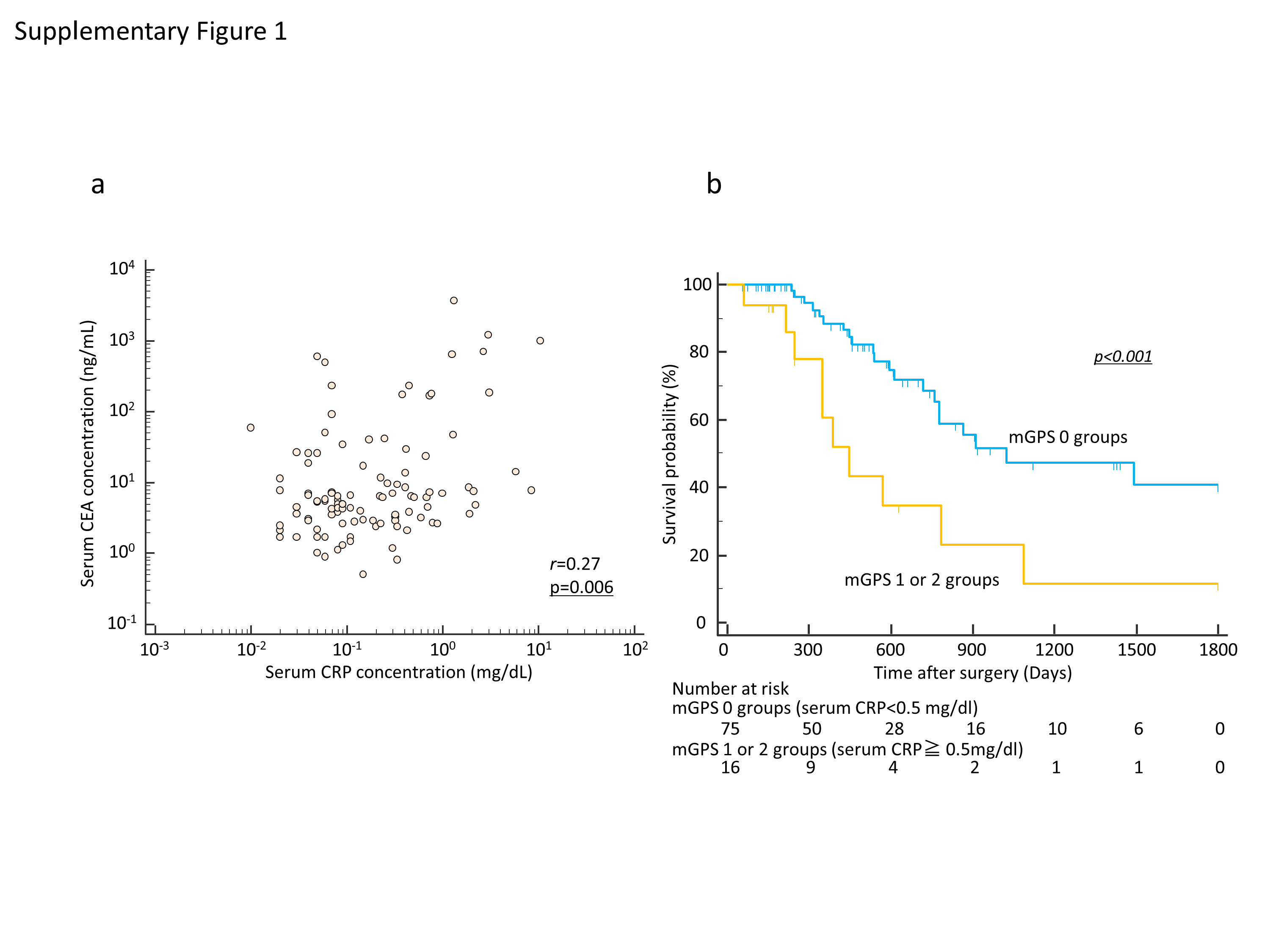


| **Supplementary Table 1** |  |  |  |  |  |  |  |  |
| --- | --- | --- | --- | --- | --- | --- | --- | --- |
| **Chronological changes in biochemical and physiological factors in GI cancer patients with or without FO-enriched nutrition** | | | | | | | | |
|  | With FO-enriched nutrition (mean ± SD) | | |  | Without FO-enriched nutrition (mean ± SD) | | |  |
|  | 0 month | 3 months | 6 months | *p value*  for trend* | 0 month | 3 months | 6 months | *p value*  for trend* |
| Alb (g/dL) | 3.7±0.4 | 3.7±0.5 | 3.8±0.6 | 0.75 | 4.0±0.4 | 3.9±0.4 | 3.9±0.5 | 0.06 |
| CRP (mg/dL) | 0.7±1.2 | 0.6±1.0 | 0.7±1.4 | 0.26 | 0.7±1.7 | 0.5±1.0 | 1.3±3.5 | ***0.049*** |
| CEA (ng/mL) | 76.5±216 | 126±361 | 89.6±210 | ***0.003*** | 105±444 | 70.5±300 | 166±722 | 0.06 |
| CA19-9 (U/mL) | 721±1738 | 152±308 | 4550±19974 | ***0.046*** | 9972±77790 | 19071±138197 | 13011±59593 | ***0.009*** |
| BMI | 21.2±2.9 | 21.1±2.8 | 20.9±3.0 | 0.39 | 21.7±3.4 | 21.9±3.5 | 22.0±3.5 | 0.73 |
| ECW/TBW | 0.4±0.01 | 0.4±0.002 | 0.4±0.01 | 0.74 | 0.4±0.01 | 0.4±0.01 | 0.4±0.01 | 0.28 |
| Skeletal muscle mass | 22.6±4.3 | 22.6±4.2 | 23.3±4.3 | ***0.0002*** | 22.5±5.0 | 22.8±5.0 | 22.7±4.8 | 0.26 |
| Lean body mass | 42.0±7.1 | 42.4±7.0 | 44.3±7.2 | ***<0.0001*** | 42.0±8.4 | 42.7±8.3 | 42.5±8.1 | 0.19 |
| Body fat quantity | 11.6±6.0 | 11.0±6.0 | 12.1±6.2 | 0.57 | 13.9±6.5 | 14.2±6.9 | 14.8±7.4 | 0.56 |
| Body fat percentage | 22.8±8.8 | 20.7±8.7 | 21.4±8.8 | 0.41 | 24.0±8.2 | 23.9±8.4 | 24.9±8.9 | 0.32 |
| Alb: Albumin, CRP: C-reactive protein, CEA: Carcinoembryonic antigen, ECW: Extracellular water, TBW: Total body water, *Friedman test | | | | | | | | |

| **Supplementary Table 2** |  |  |  |  |  |  |  |
| --- | --- | --- | --- | --- | --- | --- | --- |
| **Multivariate analysis to identify predictors of overall survival in GI cancer patients without FO-enriched nutrition** | | | | | | | |
| Variables | Univariate | | |  | Multivariate | | |
|  | HR | 95%CI | *p value* |  | HR | 95%CI | *p value* |
| Gender (male) | 0.75 | 0.35-1.63 | 0.47 |  | 0.55 | 0.24-1.24 | 0.15 |
| Age (≧70 years old)^#^ | 0.48 | 0.24-0.96 | 0.039 |  | 0.57 | 0.26-1.23 | 0.15 |
| Type of gastrointestinal cancer | 1.24 | 0.85-1.8 | 0.27 |  | 1.29 | 0.85-1.97 | 0.23 |
| UICC TNM stage classification | 1.03 | 0.61-1.75 | 0.91 |  | 1.06 | 0.62-1.8 | 0.83 |
| High serum CRP levels (≧0.5 mg/dl) | 3.27 | 1.58-6.8 | **0.0015*** |  | 2.88 | 1.26-6.54 | **0.01*** |
| ^#^ The median age at surgery was 70 years in this cohort. HR: Hazard ratio | | | |  |  | *** *p*<0.05** | |
